# Chemodiversity and Antinociceptive Activity of *Amorpha fruticosa* L. Essential Oil

**DOI:** 10.3390/plants13213045

**Published:** 2024-10-30

**Authors:** Miljana R. Đorđević Zlatković, Nikola M. Stojanović, Dragan B. Zlatković, Pavle J. Randjelović, Niko S. Radulović

**Affiliations:** 1 Department of Chemistry, Faculty of Sciences and Mathematics, University of Niš, 18000 Niš, Serbia; miljana.djordjevic@pmf.edu.rs (M.R.Đ.Z.); dragan.zlatkovic@gmail.com (D.B.Z.); 2 Department of Physiology, Faculty of Medicine, University of Niš, 18000 Niš, Serbia; nikola.st90@yahoo.com (N.M.S.); pavleus@gmail.com (P.J.R.)

**Keywords:** *Amorpha fruticosa*, essential oil, antinociceptive activity, clustering analysis

## Abstract

An essential oil dominated by germacrene D (19.3% by GC) was isolated from the fresh fruit of *Amorpha fruticosa* L. (Fabaceae). Agglomerative clustering and *k*-means clustering were employed to compare the composition of the oil with the existing literature data, suggesting that the *A. fruticosa* used in this study represents a new chemotype. The essential oil was evaluated for its antinociceptive activity using the acetic acid-induced writhing test in rats at doses of 400, 200, and 100 mg/kg. All tested doses reduced the number of writhes induced by the intraperitoneal injection of acetic acid. The 400 mg/kg dose of the oil demonstrated a 54.4% inhibition, which was statistically different from the positive control, aspirin, which showed 90.2% inhibition at a dose of 200 mg/kg. Since the injection of acetic acid produces the release of prostaglandins, such as PGE2α and PGF2α, as well as sympathetic nervous system mediators in peritoneal fluids, the results suggest that the inhibition of prostaglandin release might represent one of the possible mechanisms of action exerted by the oil.

## 1. Introduction

*Amorpha fruticosa* L. (Fabaceae) is a perennial deciduous shrub native to North America [1], known as (desert) false indigo-bush, which is related to the application of *A. fruticosa* as a dye source [2,3]. It was introduced to Europe as an ornamental plant (in the early 1700s); today, it grows naturalized outside of its native range, mostly near river banks, and is planted to stabilize soil for erosion control [1]. *Amorpha fruticosa* is one of the most dangerous invasive species in many European countries. Therefore, its exploitation could offer an affordable resource of potentially valuable chemicals. For example, its fruits are used in the perfumery and pharmaceutical industries in Bulgaria [3,4].

Traditionally, false indigo-bush has been used in Chinese medicine to treat hypertension, hematomas, contusions, burns, eczema, stomach pain, and neuralgia, which suggests an application of its secondary metabolites for medicinal purposes [5,6,7]. For a comprehensive overview of the (ethno)medicinal and non-medicinal uses of *A. fruticosa*, we refer readers to the review by Grabić et al. [7].

Previous phytochemical studies of this species resulted in the identification of family-marker classes of compounds such as isoflavonoids and their derivatives rotenoids [8,9,10,11], flavanones [12,13,14], and isoflavones [15], and prenylated stilbenoids [16]. Some metabolites of this plant are rare or unique to the false indigo-bush. The composition of the essential oils from *A. fruticosa* fruits and flowers has been investigated on several occasions. Depending on the location of plant material collection and the specific plant part subjected to analysis, the major components of the oils differed markedly. The essential oil from the fruits contained *γ*-muurolene and *ar*-curcumene, or *δ*-cadinenes, *α*-zingiberene and *α*-eudesmol, in samples from Bulgaria and Romania, respectively [5,17]. *α*-Eudesmol, *β*-eudesmol, *δ*-cadinene and (*E*)-nerolidol or *α*-pinene and myrcene were the most abundant in the flower and ripe fruit oils from Poland; the leaf oil contained *α*-eudesmol, (*E*)-*β*-ocimene, and *α*-pinene [18]. All these studies reported high yields of isolated oil (up to 1.8% for dried fruit, [5]). *γ*-Amorphene, a sesquiterpenoid isolated from this species from the first time, has been present only in small amounts in samples collected in Ukraine [19].

Several of the identified compounds from *A. fruticosa* have demonstrated significant antimicrobial activity [20], as well as NF *κ*B- and neuraminidase, proliferative [2] and TNF-*α*-inhibitory [13] effects. Extracts of *A. fruticosa* have also been shown to possess antidiabetic activity in diet-induced obese and db/db mice via the activation of the selective peroxisome PPARγ [21]. This activity was attributed to the amorfrutins present in this plant species. The exceptional properties of these natural products could potentially lead to their future biomedical application, either as lead compounds in drug discovery or as reference compounds in pharmacological research [16]. In addition to these activities, Wu and coworkers [16] found a large number of rotenoid glycosides with potent cytotoxic action against MCF-7 and HCT-116 cells. 

Considering the abundance of *A. fruticosa* in many regions, its high content of volatile compounds (and thus the potential to obtain large quantities of fruit essential oil from foraged plant material), and the fact that the pharmacological properties of *A. fruticosa* essential oil have been investigated in only two studies (antimicrobial [5] and cytotoxic [22]), the aims of this work were:

(1) Detailed chemical analysis of *A. fruticosa* fruit essential oil (from Serbia) by GC and GC-MS (gas chromatography–mass spectrometry).
(2) Statistical chemotypification of the *A. fruticosa* essential oil using clustering analysis, based on current and previously published data.
(3) An investigation of the antinociceptive activity of the oil in the acetic acid-induced writhing test. Despite the extensive data on the pharmacology of *A. fruticosa*, no study to date has addressed the analgesic activity of this species.

## 2. Results and Discussion

### 2.1. Chemical Composition of A. fruticosa Essential Oil

The list of identified volatile constituents of *A. fruticosa* fruits collected in Niš, Serbia, is compiled in Table 1. GC and GC-MS analyses enabled the identification and quantification of 105 components, comprising 92.9% of the total GC peak area detected. The sesquiterpenoid fraction was the most prominent, accounting for 83.8% of the essential oil, unevenly distributed between hydrocarbons (69.8%) and oxygenated derivatives (14.0%), followed by monoterpenoids (8.9%). The main constituents of the essential oil were germacrene D (19.3%), α-zingiberene (9.9%), *δ*-amorphene (7.6%), *γ*-muurolene (6.5%), (*E*)-caryophyllene (5.4%), and *α*-eudesmol (4.5%). These results align with most existing studies on fruit essential oil [5,16,17], which also reported sesquiterpene hydrocarbons as the main constituents of *A. fruticosa* fruit essential oil. The only outlier was the study by Lis and Góra [18], which identified α-pinene (ca. 20%) as the main constituent in the monoterpene-dominated essential oil from Polish *A. fruticosa*. The observed yield (1.3%, *w*/*w*) was significantly higher than that previously reported for fresh fruits by Lis and Góra (0.45%, [18]). 

One major difference we immediately observed was that the major component of our oil, germacrene D, was present in levels ≤ 10% [17] or entirely absent in other studies of *A. fruticosa* fruit oil (see amorpha_fruticosa_dataset.csv in the Supplementary Materials). The implications of this discrepancy are further discussed in Section 2.2, where we provided statistical evidence strongly suggesting that the *A. fruticosa* examined in this study represents a previously unreported chemotype.

### 2.2. Clustering Analysis

**Figure 1:**
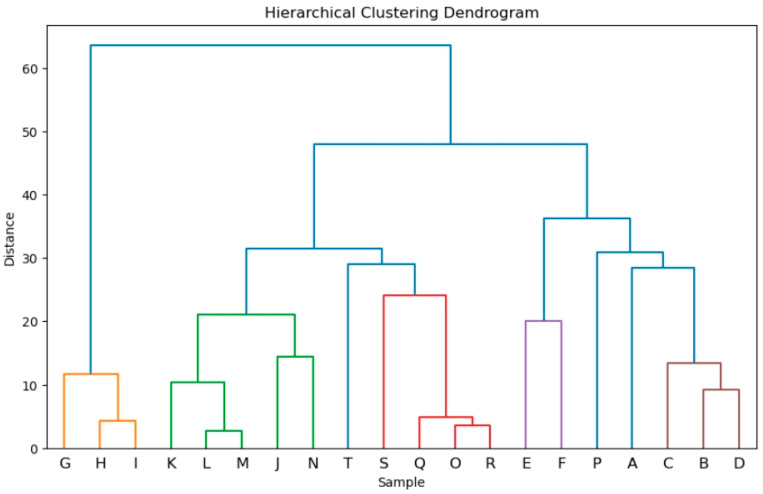
Hierarchical clustering dendrogram of *A. fruticosa* essential oil samples based on their chemical composition. The color threshold was set at 25. Different colors correspond to different clusters. For details on the sample labels, see Table 2.

**Figure 2:**
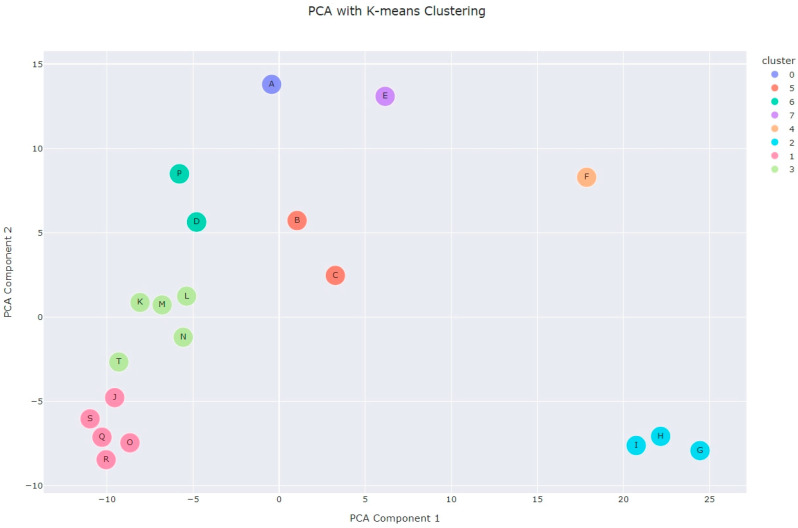
Principal component analysis (PCA) scatter plot of *A. fruticosa* essential oil samples with *k*-means clustering. The plot shows the distribution of samples along the first two principal components, colored by their respective clusters. For details on the sample labels, see Table 2.

A statistical comparison of *A. fruticosa* essential oil from our study with composition data reported by other authors was performed using *k*-means clustering (following principal component analysis) and agglomerative clustering with Euclidean distances. The results of the analyses are shown in Figure 1 and Figure 2 (the origin of each sample is listed in Table 2). Both methods yielded similar results (see amorpha.html in SI for complete cluster assignments). As expected, samples from the same studies that employed plant material of the same origin mostly grouped into one cluster. This is clearly visualized in the hierarchical clustering dendrogram (Figure 1). Samples I, H, and G (fruit essential oil from Lis and Góra, 2001 [18]; fruit differing in ripeness and freshness) are quite separated from any other sample. Samples J, K, L, M, and N from Stoyanova et al. [17] (different storage times of the fruit) also formed a distinct cluster. Samples B, C, and D from Ivănescu et al. [5] were all collected in Iaşi, Romania, and differed only in their collection dates. These observations were expected, as the differences in the handling of the plant materials were not drastic and did not significantly alter the composition of the oil.

Samples E and F (the essential oils of the flowers and leaves, respectively), as expected, statistically differed from any other sample. However, some observations were unexpected. Samples O, Q, and R from Chen et al. [16] ended up in the same cluster, but sample P was quite different, as shown by both clustering methods (Figure 1 and Figure 2). The additional liquid–liquid separation column used for obtaining sample P (compared to sample O) seems to have significantly changed its composition. Sample S from Marinas et al. [22] is very closely related to the sample from Chen et al.

Based on these observations, sample A, obtained in this study, is not related to any of the previously reported samples. Although the analysis shows it is closest to samples B-D from Ivanescu et al. [5], germacrene D, which is the major constituent in our oil, was not detected in four samples from Romania. To better understand the differences between the obtained clusters, we also performed cluster characterization (post-clustering process where centroids or features within each cluster are analyzed to understand which variables are driving the formation of the clusters). For more details, see Cell 11 in amorpha.html, Supplementary Materials. With all this in mind, it is our opinion that the *A. fruticosa* used in this study represents a unique (germacrene D-driven) chemotype, which is distinctive from three other chemotypes:

1. Monoterpene-dominated chemotype [18], major cluster contributors: α-pinene (centroid value = 21.6) and myrcene (16.6);
2. Sesquiterpene-dominated chemotype I [16,17]; in our opinion, the *A. fruticosa* used in these two studies are of the same chemotype, and the difference in the clusters is caused only by different drying periods: samples K-N were obtained from fruit that was dried for over 6 months, while other samples were from fruit dried for a shorter period of time. Major cluster contributors: δ-cadinene, γ-muurolene, γ-cadinene;
3. Sesquiterpene-dominated chemotype II [5], similar to chemotype A but with lower levels of δ- and γ-cadinene and higher contribution of α-zingiberene and α-eudesmol.

The results of the clustering analysis supported our decision to use fresh *A. fruticosa* fruits in this study. Stoyanova et al. [17] reported that the volatile profile of this plant species varies depending on the storage period, and this effect can be clearly observed in the PCA scatter plot (Figure 2), where the essential oil from samples stored for longer periods (>6 months, K, L, M, N) forms a distinct cluster compared to the oil from fresh fruits (J). Thus, we chose to use essential oil from fresh fruits for two main reasons:

(1) The profile of fresh material closely reflects the state of the plant at the time of collection. This is especially important for future studies dealing with the discovery of new chemotypes.
(2) Another key reason was to ensure the reproducibility of the study. Any biological activity observed for *A. fruticosa* essential oil would be more reproducible when fresh fruit is used. Storage or drying introduces variability (e.g., loss of monoterpenes, oxidation), which is difficult to control. Even attempts to replicate the storage process would need to account for factors such as temperature and humidity. These variables are eliminated when fresh material is used.

### 2.3. Antinociceptive Activity

**Figure 3:**
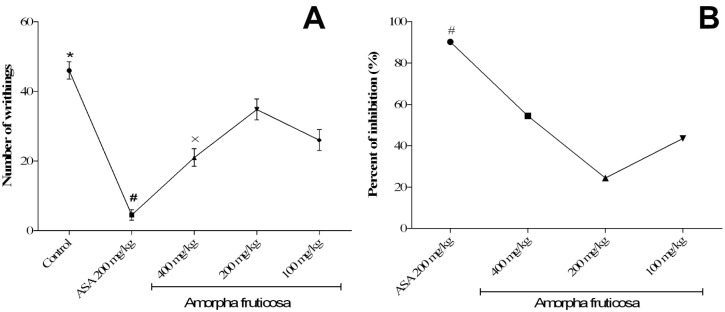
Effect of acute administration of *A. fruticosa* essential oil in doses of 100, 200, and 400 mg/kg i.p. on the number of writhes in mice induced by acetic acid. Values are mean ± SD, one-way ANOVA followed by Tukey’s test; * *p* < 0.01 vs. ASA and essential oil in all doses; # *p* < 0.01 vs. vehicle and essential oil in all doses; × *p* < 0.01 vs. *A. fruticosa* (100 and 200 mg/kg). (**A**): number of writhings vs. concentration; (**B**): percent of inhibition vs. concentration.

The essential oil demonstrated strong-to-moderate antinociceptive activity in the chemically induced pain model (Figure 3). A statistically significant difference in the degree of activity was found between the groups of animals treated with all doses of the essential on one side and vehicle/ASA-treated animals on the other (*p* < 0.01). Additionally, the calculated percentage of abdominal writhing inhibition for the 400 mg/kg dose of the oil (54.4%) statistically differed from the positive control, aspirin (90.2%).

The injection of acetic acid triggers the release of prostaglandins, such as PGE_2α_ and PGF_2α_, along with sympathetic nervous system mediators in the peritoneal fluid, causing a cascade of events that lead to abdominal contortions due to inflammation [24]. This test is nonspecific, since numerous drugs acting on different receptor/enzyme systems can reduce the number of acetic acid-induced writhes [25]. Amorfrutin A, a natural compound found in *A. fruticosa* fruits, has been shown to act as a nuclear factor-κB inhibitor (via different mechanisms), providing evidence for its potential usage in pathological conditions that involve inflammation. However, we did not detect it and it is very unlikely that it could be an essential oil component [26]. 

Our essential oil possessed significant amounts of *α*-pinene (>4.2%), which was proven to significantly reduce the number of writhes induced by acetic acid in a dose of 200 mg/kg [27]. However, several other constituents, such as germacrene D, caryophyllene, zingiberene, *δ-* and *γ*-cadinenes, and others (Table 1) may also contribute to the overall activity of the essential oil.

## 3. Conclusions

The GC and GC-MS analyses of *A. fruticosa* fruit essential oil from Serbia led to the identification of 105 constituents, with germacrene D being the major compound. Clustering analysis suggests that the *A. fruticosa* used in this study represents a distinct new (germacrene D-dominated) chemotype and differs from the three previously reported chemotypes (one monoterpenoid-dominated and two sesquiterpenoid-dominated types). The statistical analysis also confirmed that fruit storage can affect essential oil composition, and prolonged storage could even result in the false identification of new chemotypes.

For this reason, we believe future studies on *A. fruticosa* essential oil should focus on using fresh plant material to ensure the reproducibility of biological assays, as the oil composition directly influences the outcomes of these assays. Changes in oil composition due to storage could affect the biological activities. Additionally, the hydrodistillation of fresh material prevents the loss of volatile compounds and ensures higher oil yields. Future research should also explore whether different hydrodistillation methods (e.g., microwave-assisted hydrodistillation, CO_2_ extraction, etc.) can further increase yields.

The essential oil exhibited strong-to-moderate antinociceptive activity in a chemically induced pain model. Given the high yield of oil and the wide geographical distribution of this plant species, we believe that *A. fruticosa* essential oil has significant phytopharmacological potential. Further research is needed to identify the compounds responsible for this activity.

## 4. Materials and Methods

### 4.1. GC and GC-MS

The GC-MS analyses were performed on a Hewlett-Packard 6890N gas chromatograph equipped with a fused silica capillary column DB-5MS (5% diphenylsiloxane, 95% dimethylsiloxane, 30 m × 0.25 mm, film thickness 0.25 μm; Agilent Technologies, Palo Alto, CA, USA) and coupled with a 5975B mass-selective detector from the same company. The injector and interface temperatures were set to 250 °C and 320 °C, respectively. The oven temperature was programmed to increase from 70 to 290 °C at a rate of 5 °C/min, followed by an isothermal hold for 10 min. Helium was used as the carrier gas at a flow rate of 1.0 mL/min. A sample (10 μL of the oil in 1 mL Et_2_O) was injected in pulsed-split mode (with a flow rate of 1.5 mL/min for the first 0.5 min, then 1.0 mL/min for the remainder of the analysis; split ratio 40:1). The MS conditions were as follows: ionization voltage of 70 eV, acquisition mass range of 35–500 amu, and a scan time of 0.34 s. The GC (FID) analyses were performed under the same experimental conditions using the same column as described for GC-MS. The percentage composition was calculated from the GC peak areas without the use of correction factors. Linear retention indices were calculated for all identified components using standards of *n*-alkanes (C_7_–C_17_). AMDIS (v. 2.70) software was used for chromatogram deconvolution and mass spectral libraries (Wiley 7, NIST 14, MassFinder 2.3, and Adams library [28]) were searched with NIST MS Search software (v. 2.0).

### 4.2. Plant Material

Plant material (ripe fruits) of *A. fruticosa* was collected in September 2011 near the Nišava River in Niš, Serbia (exact coordinates: 43°19′13″ N, 21°56′27″ E). A voucher specimen was deposited with the Herbarium Collection of the Faculty of Sciences and Mathematics, University of Niš, under acquisition number 18661. The identity of the material was confirmed by the curator of the herbarium. 

### 4.3. Chemicals

All solvents (HPLC-grade) were purchased from Sigma-Aldrich (St. Louis, MO, USA). Authentic chemical samples were obtained from Merck (Darmstadt, Germany), and Carl Roth (Karlsruhe, Germany) in the highest available purity. For the determination of retention indices, a commercial *n*-alkane mixture (Sigma-Aldrich, St. Louis, MO, USA) ranging from heptane to eicosane (C_7_–C_40_) was used.

### 4.4. Extraction of Essential Oils

Fresh fruits (260 g) of *A. fruticosa* were homogenized in a blender and submitted to hydrodistillation with approximately 2.5 L of H_2_O for 3 h, using an original *Clevenger*-type apparatus. The obtained oil was separated by extraction with Et_2_O and dried with anhydrous MgSO_4_. The solvent was evaporated under a gentle stream of N_2_ at room temperature, to exclude any loss of the essential oil, and the sample was immediately analyzed. To determine the oil yield, after most of the Et_2_O was removed under a stream of N_2_, the residue was exposed to a vacuum at room temperature for a short time to eliminate any remaining solvent. The yield of the obtained oil (3.5 g) was 1.3% (*w*/*w*). The essential oil was stored in a freezer at −20 °C without any further treatment. 

### 4.5. Clustering Analysis

The dataset used for the statistical analysis (amorpha_fruticosa_dataset.csv, Supplementary Materials) was compiled from composition data for essential oils labeled A-Q (see Table 2 for the origin of each sample). Constituents that occurred above the 1% level were filtered and used for the analysis. All analyses were performed using the scikit-learn package in Python 3.10.13 [29] within the Jupyter Lab (v. 4.0.9) environment [30]. Visualization was performed using the matplotlib and Plotly packages. The complete code is provided in the Supplementary Materials in both.ipynb and .html formats.

Prior to analysis, the dataset was transposed to facilitate clustering. Hierarchical clustering was conducted using agglomerative clustering with Euclidean distance as the metric. The pairwise distance matrix was computed using the *pdist* function, and hierarchical clustering was performed using the Ward method to generate a linkage matrix. A dendrogram was created to visualize the hierarchical clustering, with a color threshold set at 25. Based on this dendrogram, eight clusters were selected for agglomerative clustering. Samples within each cluster were identified and listed.

Principal component analysis (PCA) was used to reduce the dimensionality of the dataset to two principal components. *K*-means clustering was applied to the PCA-transformed data. The optimal number of clusters was determined using the Elbow Method, which involved plotting the inertia values for a range of clusters (K = 1 to 10). Based on visual observation, eight clusters were selected for *k*-means clustering. The results were visualized using a scatter plot created with Plotly Express (v. 5.24.1) [31], where the two PCA components were plotted, and points were colored according to their cluster assignment.

### 4.6. Animals and Treatment

The adult male and female Wistar rats (200–250 g) used in this experiment were housed in our facilities (Vivarium of the Institute of Biomedical Research, Medical Faculty, University of Niš, Niš, Serbia). They were kept in groups of 6 animals per cage, maintained in standard laboratory conditions at 22 ± 2 °C and 60% humidity, with food and water available ad libitum. All animals fasted for 12 h, although they were still allowed free access to water, before the commencement of the experiments. All rats were individually weighed, and the essential oil of *A. fruticosa* L. was applied at doses of 400, 200, and 100 mg/kg dissolved in olive oil, while the negative and positive control groups received the vehicle (olive oil in a dose of 10 mL/kg) and aspirin (ASA; in a dose of 200 mg/kg), respectively. All substances were administered intraperitoneally (i.p.) to animals 1 h before the test.

The experiments were performed in accordance with the Declaration of Helsinki and European Community guidelines for the ethical handling of laboratory animals (EU Directive of 2010; 2010/63/EU), and the experimental protocols were commenced after being approved by the institutional animal ethics committee (No. 01-4097-2 from 2011).

### 4.7. Acetic Acid-Induced Abdominal Writhing

The method adopted in this study was previously described by Radulović et al. [24]. Briefly, one hour after the administration of all substances, 1% (*v*/*v*) acetic acid (500 μL) was injected i.p. into all animals. During the next 20 min, specific contractions of the abdominal muscles, described as writhing, were counted. The % of inhibition was calculated as follows:

$$\%\;\mathrm{inhibition}=100\times \frac{\mathrm{number}\;\mathrm{of}\;\mathrm{writhes}\;\left(\mathrm{control}\right)-\mathrm{number}\;\mathrm{of}\;\mathrm{writhes}\;\left(\mathrm{test}\right)}{\mathrm{number}\;\mathrm{of}\;\mathrm{writhes}\;\left(\mathrm{control}\right)}$$

### 4.8. Statistical Analysis

Data obtained from the experiments are presented as mean ± SD and were further analyzed using one-way ANOVA followed by Tukey’s post hoc test (GraphPad Prism version 5.03, San Diego, CA, USA). Probability values (*p*) less than 0.05 were considered statistically significant.

## Figures and Tables

**Table 1:** Chemical composition of the essential oil isolated from fruits of *A. fruticosa*.

No. ^a^	Compound	RI ^b^	Content [%] ^c^	Compound Class ^d^	Identification Method ^e^
1	1-Pentanol	764	tr	O	MS, *RI*, CoI
2	Hexanal	801	0.2	O	MS, *RI*, CoI
3	1-Hexanol	863	tr	O	MS, *RI*, CoI
4	Heptanal	901	tr	O	MS, *RI*, CoI
5	*α*-Thujene	921	tr	MT	MS, *RI*
6	*α*-Pinene	930	4.2	MT	MS, *RI*, CoI
7	Camphene	948	tr	MT	MS, *RI*, CoI
8	(*E*)-2-Heptenal	948	tr	O	MS, *RI*
9	Benzaldehyde	959	tr	O	MS, *RI*, CoI
10	Sabinene	969	tr	MT	MS, *RI*
11	1-Octen-3-ol	970	tr	O	MS, *RI*
12	*β*-Pinene	977	0.7	MT	MS, *RI*, CoI
13	Myrcene	982	1.0	MT	MS, *RI*
14	2-Pentylfuran	983	tr	O	MS, *RI*
15	*α*-Phellandrene	1005	tr	MT	MS, *RI*
16	*α*-Terpinene	1015	tr	MT	MS, *RI*, CoI
17	*o*-Cymene	1021	0.1	MT	MS, *RI*, CoI
18	Limonene	1026	0.3	MT	MS, *RI*, CoI
19	(*Z*)-*β*-Ocimene	1028	1.4	MT	MS, *RI*, CoI
20	1,8-Cineole	1030	tr	MT *	MS, *RI*, CoI
21	(*E*)-*β*-Ocimene	1039	0.2	MT	MS, *RI*, CoI
22	Phenylacetaldehyde	1039	tr	O	MS, *RI*, CoI
23	*γ*-Terpinene	1053	0.1	MT	MS, *RI*, CoI
24	*cis*-Sabinene hydrate	1066	tr	MT	MS, *RI*
25	Terpinolene	1083	tr	MT	MS, *RI*, CoI
26	Linalool	1094	0.3	MT *	MS, *RI*, CoI
27	*trans*-Sabinene hydrate	1100	0.1	MT	MS, *RI*, CoI
28	Nonanal	1100	tr	O	MS, *RI*, CoI
29	Isopentyl isovalerate	1102	tr	O	MS, *RI*, CoI
30	*trans*-Pinocarveol	1139	tr	MT *	MS, *RI*
31	*trans*-Verbenol	1143	tr	MT *	MS, *RI*
32	Isopulegol	1146	tr	MT *	MS, *RI*
33	*iso*-Isopulegol	1156	tr	MT *	MS, *RI*
34	(*E*)- 2-Nonen-1-ol	1164	tr	O	MS, *RI*
35	Terpinen-4-ol	1175	0.2	MT *	MS, *RI*, CoI
36	*α*-Terpineol	1192	tr	MT *	MS, *RI*, CoI
37	Citronellol	1220	0.3	MT *	MS, *RI*, CoI
38	(2*E*,4*E*)-2,4-Decadienal	1314	tr	O	MS, *RI*
39	*α*-Cubebene	1343	0.7	ST	MS, *RI*
40	*α*-Ylangene	1366	0.7	ST	MS, *RI*
41	*α*-Copaene	1372	2.6	ST	MS, *RI*
42	*β*-Bourbonene	1381	tr	ST	MS, *RI*
43	7-*epi*-Sesquithujene	1382	1.2	ST	MS, *RI*
44	*β*-Cubebene	1384	tr	ST	MS, *RI*
45	*β*-Elemene	1385	1.1	ST	MS, *RI*
46	Sesquithujene	1396	0.1	ST	MS, *RI*
47	*α*-Gurjunene	1404	0.5	ST	MS, *RI*
48	*cis*-*α*-Bergamotene	1408	tr	ST	MS, *RI*
49	*β*-Ylangene	1415	tr	ST	MS, *RI*
50	(*E*)-Caryophyllene	1417	5.4	ST	MS, *RI*, CoI
51	*β*-Copaene	1427	1.4	ST	MS, *RI*
52	*trans*-*α*-Bergamotene	1428	tr	ST	MS, *RI*
53	Aromadendrene	1435	0.9	ST	MS, *RI*
54	Cadina-3,5-diene	1445	tr	ST	MS, *RI*
55	(*E*)-*β*-Farnesene	1446	0.5	ST	MS, *RI*
56	*α*-Humulene	1452	1.7	ST	MS, *RI*, CoI
57	*cis*-Cadina-1(6),4-diene	1456	tr	ST	MS, *RI*
58	*cis*-Muurola-4(14),5-diene	1458	0.3	ST	MS, *RI*
59	*trans*-Cadina-1(6),4-diene	1468	0.5	ST	MS, *RI*
60	*γ*-Muurolene	1471	6.5	ST	MS, *RI*
61	*ar*-Curcumene	1476	tr	ST	MS, *RI*
62	Germacrene D	1479	19.3	ST	MS, *RI*
63	(*Z*,*E*)-*α*-Farnesene	1484	tr	ST	MS, *RI*
64	*β*-Selinene	1486	1.3	ST	MS, *RI*
65	α-Zingiberene	1489	9.9	ST	MS, *RI*
66	*α*-Muurolene	1494	tr	ST	MS, *RI*
67	(*E*,*E*)-*α*-Farnesene	1497	tr	ST	MS, *RI*
68	*β*-Bisabolene	1502	tr	ST	MS, *RI*
69	*γ*-Cadinene	1509	4.2	ST	MS, *RI*
70	Cubebol	1511	tr	ST *	MS, *RI*
71	*δ*-Amorphene	1514	7.6	ST	MS, *RI*
72	*trans*-Calamenene	1516	tr	ST	MS, *RI*
73	*β*-Sesquiphellandrene	1518	2.3	ST	MS, *RI*
74	(*E*)-*γ*-Bisabolene	1521	tr	ST	MS, *RI*
75	10-*epi*-Cubebol	1526	0.1	ST *	MS, *RI*
76	*trans*-Cadina-1,4-diene	1528	0.6	ST	MS, *RI*
77	*α*-Cadinene	1532	0.5	ST	MS, *RI*
78	*α*-Calacorene	1537	tr	ST	MS, *RI*
79	*cis*-Sesquisabinene hydrate	1537	0.7	ST *	MS, *RI*
80	Elemol	1543	0.9	ST *	MS, *RI*
81	(*E*)-Nerolidol	1553	0.3	ST *	MS, *RI*
82	*β*-Calacorene	1557	tr	ST	MS, *RI*
83	1*α*,10*α*-Epoxyamorph-4-ene	1565	tr	ST *	MS, *RI*
84	Germacrene D-4-ol	1572	tr	ST *	MS, *RI*
85	Spathulenol	1573	2.0	ST *	MS, *RI*
86	Caryophyllene oxide	1579	0.5	ST *	MS, *RI*, CoI
87	Salvial-4(14)-en-1-one	1588	tr	ST *	MS, *RI*
88	Globulol	1591	0.6	ST *	MS, *RI*
89	Ledol	1601	tr	ST *	MS, *RI*
90	Rosifoliol	1603	tr	ST *	MS, *RI*
91	Humulene epoxide II	1606	tr	ST *	MS, *RI*
92	Zingiberenol	1608	0.3	ST *	MS, *RI*
93	1,10-di-*epi*-Cubenol	1611	tr	ST *	MS, *RI*
94	Muurola-4,10(14)-dien-1*β*-ol	1621	tr	ST *	MS, *RI*
95	1-*epi*-Cubenol	1623	0.3	ST *	MS, *RI*
96	Eremoligenol	1626	tr	ST *	MS, *RI*
97	*γ*-Eudesmol	1627	1.3	ST *	MS, *RI*
98	*epi*-*α*-Cadinol (syn. τ-cadinol)	1637	1.2	ST *	MS, *RI*
99	*epi*-*α*-Murrolol (syn. τ-muurolol)	1639	tr	ST *	MS, *RI*
100	*α*-Muurolol (syn. torreyol)	1642	0.2	ST *	MS, *RI*
101	*α*-Eudesmol	1651	4.5	ST *	MS, *RI*
102	*epi*-*β*-Bisabolol	1665	0.2	ST *	MS, *RI*
103	Cadalene	1667	tr	ST	MS, *RI*
104	*α*-Bisabolol	1679	0.9	ST *	MS, *RI*, CoI
105	Amorpha-4,9-dien-2-ol	1685	tr	ST *	MS, *RI*
	**Total identified [%]**		92.9		
	MT—Monoterpenes		8.1		
	MT *—Oxygenated monoterpenes		0.8		
	ST—Sesquiterpenes		69.8		
	ST *—Oxygenated sesquiterpenes		14.0		
	O—Other		0.2		

^a^ Compounds listed in the order of elution from a DB-5MS column. ^b^ Linear retention indices (RIs) determined experimentally on the DB-5MS column relative to a series of C_7_–C_17_ *n*-alkanes. ^c^ Values are the means of three individual analyses; tr, trace amounts (<0.05%). ^d^ The abbreviations of the compound classes are given at the end of the table. ^e^ Compound identification: RIs, retention indices matching with literature data; MS, mass spectra matching; CoI, coinjection with a pure reference compound.

**Table 2:** Labels and origin of *A. fruticosa* essential oil samples used in the clustering analysis.

Label	Reference	Description
A	Essential oil from this study	fresh ripe fruits/hydrodistillation Clevenger apparatus
B	Ivănescu et al., 2014 [5]	air-dried fruits/hydrodistillation Clevenger apparatus
C		air-dried fruits/hydrodistillation Clevenger apparatus
D		air-dried fruits/hydrodistillation Clevenger apparatus
E	Lis and Góra, 2001 [18]	fresh flowers/hydrodistillation
F		fresh leaves/hydrodistillation
G		fresh crushed unripe fruits/hydrodistillation
H		fresh crushed ripe fruits/hydrodistillation
I		air-dried crushed ripe fruits/hydrodistillation
J	Stoyanova et al., 2003 [17]	air-dried crushed fruits stored for 0–6 months/hydrodistillation
K		air-dried crushed fruits stored for 6 months/hydrodistillation
L		air-dried crushed fruits stored for 18 months/hydrodistillation
M		air-dried crushed fruits stored for 30 months/hydrodistillation
N		air-dried crushed fruits stored for 40 months/hydrodistillation
O	Chen et al., 2017 [16]	air-dried crushed fruits/MHD-LLE *
P		air-dried crushed fruits/MHD-LLE **
Q		air-dried crushed fruits/microwave-assisted hydrodistillation
R		air-dried crushed fruits/hydrodistillation Clevenger apparatus
S	Marinas et al., 2021 [22]	air-dried crushed fruits/hydrodistillation Clevenger apparatus
T	Kozuharova et al., 2020 [23]	air-dried fruits/hydrodistillation Clevenger apparatus

* MHD-LLE—microwave-assisted hydrodistillation concatenated liquid–liquid extraction procedure—essential oil in first separation column; ** microwave-assisted hydrodistillation concatenated liquid–liquid extraction procedure—essential oil in second separation column.

## Data Availability

Data are contained within the article and Supplementary Materials.

## Supplementary Materials

The following supporting information can be downloaded at: https://www.mdpi.com/article/10.3390/plants13213045/s1, amorpha_fruticosa_dataset.csv (dataset used for the clustering analysis), amorpha.ipynb (Jupyter notebook with Python code), and amorpha.html (amorpha.ipynb converted to .html format so it can be viewed in a browser).
