# Supplementary material for: Chemodiversity and Antinociceptive Activity of Amorpha fruticosa L. Essential Oil
Source: Plants (Basel). 2024 Oct 30;13(21):3045. doi: 10.3390/plants13213045 (PMC11548152; doi:10.3390/plants13213045)
Supplement: Supplementary file 1 [file plants-13-03045-s001.zip › plants-3251492-supplementary/amorpha.html]

amorpha


In [1]:

```
import os
os.environ["OMP_NUM_THREADS"] = '1'
# Set the environment variable to avoid memory leak on Windows with MKL
```

In [2]:

```
import pandas as pd
from sklearn.cluster import KMeans
import matplotlib.pyplot as plt

# The dataset file should be in the same folder as the notebook file
INPUT_FILE = "amorpha_fruticosa_dataset.csv"
```

In [3]:

```
!pip install plotly
```

```
Requirement already satisfied: plotly in c:\users\dragan\appdata\local\programs\python\python312\lib\site-packages (5.24.0)
Requirement already satisfied: tenacity>=6.2.0 in c:\users\dragan\appdata\local\programs\python\python312\lib\site-packages (from plotly) (9.0.0)
Requirement already satisfied: packaging in c:\users\dragan\appdata\local\programs\python\python312\lib\site-packages (from plotly) (24.1)
```

In [4]:

```
amorpha_dataset = pd.read_csv(INPUT_FILE, index_col=0)

# Transpose the dataset
amorpha_dataset = amorpha_dataset.T
```

In [5]:

```
from sklearn.cluster import AgglomerativeClustering
from scipy.spatial.distance import pdist
from scipy.cluster.hierarchy import linkage, dendrogram

data = amorpha_dataset.copy()

# Calculate the pairwise distance matrix using Euclidean distance
distance_matrix = pdist(data, metric='euclidean')

# Perform hierarchical clustering using the linkage matrix
Z = linkage(distance_matrix, method='ward')

# Create the dendrogram to visualize the hierarchy
plt.figure(figsize=(10, 6))
dendrogram(Z, orientation='top', labels=data.index, color_threshold=25)
plt.xlabel('Sample')
plt.ylabel('Distance')
plt.title('Hierarchical Clustering Dendrogram')
plt.show()
```

In [6]:

```
# The number of clusters (8) is determined by visual analysis
num_clusters = 8

# Perform agglomerative clustering
agglomerative_clustering = AgglomerativeClustering(n_clusters=num_clusters, metric='euclidean', linkage='ward')
data['cluster'] = agglomerative_clustering.fit_predict(data)

clusters = data.groupby('cluster').apply(lambda x: x.index.tolist())
for cluster_num, samples in clusters.items():
    print(f"Cluster {cluster_num} contains samples: {samples}")
```

```
Cluster 0 contains samples: ['O', 'Q', 'R', 'S']
Cluster 1 contains samples: ['J', 'K', 'L', 'M', 'N']
Cluster 2 contains samples: ['B', 'C', 'D']
Cluster 3 contains samples: ['G', 'H', 'I']
Cluster 4 contains samples: ['E', 'F']
Cluster 5 contains samples: ['P']
Cluster 6 contains samples: ['T']
Cluster 7 contains samples: ['A']
```

In [7]:

```
from sklearn.decomposition import PCA

data = amorpha_dataset.copy()

# Reduce the data to two dimensions using PCA
pca = PCA(n_components=2)
data_pca = pca.fit_transform(data)

# Create a DataFrame with PCA data and sample names
pca_df = pd.DataFrame(data_pca, columns=['PCA Component 1', 'PCA Component 2'])
pca_df['Sample'] = data.index
```

In [8]:

```
inertia = []
for k in range(1, 11):
    kmeans = KMeans(n_clusters=k, n_init=10, random_state=42)
    kmeans.fit(data_pca)
    inertia.append(kmeans.inertia_)
plt.plot(range(1, 11), inertia)
plt.xlabel('Number of clusters (K)')
plt.ylabel('Inertia')
plt.title('Elbow Method')
plt.show()
```

In [9]:

```
# The number of clusters (8) is chosen after visual analysis of the Elbow plot
num_clusters = 8

# Perform K-means clustering on the PCA-transformed data
kmeans = KMeans(n_clusters=num_clusters, n_init=10, random_state=42)
pca_df['cluster'] = kmeans.fit_predict(data_pca)

# Print the cluster assignments
pca_df['cluster'] = pca_df['cluster'].astype(str)

clusters = pca_df.groupby('cluster').apply(lambda x: x['Sample'].tolist())
for cluster_num, samples in clusters.items():
    print(f"Cluster {cluster_num} contains samples: {samples}")
```

```
Cluster 0 contains samples: ['A']
Cluster 1 contains samples: ['J', 'O', 'Q', 'R', 'S']
Cluster 2 contains samples: ['G', 'H', 'I']
Cluster 3 contains samples: ['K', 'L', 'M', 'N', 'T']
Cluster 4 contains samples: ['F']
Cluster 5 contains samples: ['B', 'C']
Cluster 6 contains samples: ['D', 'P']
Cluster 7 contains samples: ['E']
```

In [10]:

```
import plotly.express as px

pca_df['cluster'] = pca_df['cluster'].astype(str)

fig = px.scatter(pca_df, 
                 x='PCA Component 1', 
                 y='PCA Component 2', 
                 color='cluster',
                 hover_data={'Sample': True, 'cluster': False},
                 title='PCA with K-means Clustering',
                 text='Sample',
                 size=[10] * len(pca_df))

fig.update_traces(textposition='middle center')

# Customize the plot appearance
fig.update_layout(showlegend=True, width=800, height=600, template="seaborn")

# Show the plot
fig.show()
```

In [11]:

```
# Perform K-means clustering on the original data
num_clusters = 8
kmeans = KMeans(n_clusters=num_clusters, n_init=10, random_state=42)
data['cluster'] = kmeans.fit_predict(amorpha_dataset)

# Get the centroids in the original feature space
centroids = kmeans.cluster_centers_

# Create a DataFrame for the centroids with compound names as columns
centroids_df = pd.DataFrame(centroids, columns=amorpha_dataset.columns)

# Add cluster labels to the DataFrame
centroids_df['cluster'] = range(num_clusters)

# Display the centroids to see which compounds are driving each cluster
print("Centroids in the original feature space:")
print(centroids_df)

# For each cluster, find the top contributing compounds
top_compounds_per_cluster = {}
for cluster_num in range(num_clusters):
    centroid = centroids_df.iloc[cluster_num].drop('cluster')
    # Sort by the absolute value to find compounds with the highest values
    top_compounds = centroid.abs().sort_values(ascending=False).head()
    top_compounds_per_cluster[cluster_num] = top_compounds.index.tolist()

# Print out the top contributing compounds for each cluster
print("Please notice that the cluster indices (i.e. names) might be different than in previous cell!")
for cluster_num, compounds in top_compounds_per_cluster.items():
    print(f"Cluster {cluster_num} is mainly influenced by compounds: {compounds}")

# Create a DataFrame to map samples to their clusters
samples_clusters_df = pd.DataFrame({
    'Sample': amorpha_dataset.index,
    'Cluster': data['cluster']
})

# Print out the samples belonging to each cluster
print("\nSamples in each cluster:")
for cluster_num in range(num_clusters):
    cluster_samples = samples_clusters_df[samples_clusters_df['Cluster'] == cluster_num]['Sample'].tolist()
    print(f"Cluster {cluster_num} contains samples: {cluster_samples}")
```

```
Centroids in the original feature space:
   alpha-Pinene  beta-Pinene  3-Carene    Myrcene  p-Cymene  Limonene  \
0     21.566667     1.633333      0.00  16.566667  0.000000       0.0   
1      4.900000     0.433333      0.00   2.000000  0.333333       0.8   
2      3.875000     0.000000      2.55   0.000000  0.000000       0.0   
3      0.000000     0.000000      0.00   0.000000  0.000000       0.0   
4      6.750000     0.550000      0.00   5.100000  0.000000       0.0   
5      4.980000     0.000000      0.00   0.320000  0.000000       0.0   
6      4.200000     0.000000      0.00   1.000000  0.000000       0.0   
7      0.000000     0.000000      4.20   0.000000  0.000000       0.0   

   (Z)-beta-Ocimene   1,8-Cineole  (E)-beta-Ocimene  Linalool  ...  Cubenol  \
0          7.133333  1.533333e+00      2.733333e+00  0.000000  ...     0.00   
1          1.533333  0.000000e+00      0.000000e+00  1.066667  ...     0.00   
2          0.425000  0.000000e+00      0.000000e+00  0.550000  ...     0.00   
3          0.000000  0.000000e+00      0.000000e+00  0.000000  ...     0.00   
4          3.950000  8.000000e-01      5.950000e+00  0.000000  ...     3.85   
5          0.000000 -5.551115e-17     -2.220446e-16  0.000000  ...     0.00   
6          1.400000  0.000000e+00      0.000000e+00  0.000000  ...     0.00   
7          0.000000  0.000000e+00      2.000000e+00  0.000000  ...     0.00   

   Isolongifolol methyl ether  alpha-Cadinol  gamma-Eudesmol  \
0                         0.0   0.000000e+00            0.00   
1                         0.0   1.900000e+00            1.80   
2                         0.0   0.000000e+00            1.95   
3                         7.7   0.000000e+00           15.10   
4                         0.0   0.000000e+00            3.60   
5                         0.0  -5.551115e-17            1.68   
6                         0.0   0.000000e+00            1.30   
7                         0.0   0.000000e+00            0.00   

   epi-alpha-Cadinol (=tau-cadinol)  beta-Eudesmol  alpha-Eudesmol  \
0                      1.110223e-16       1.766667           3.000   
1                      1.110223e-16       0.000000           6.700   
2                      2.500000e-01       0.000000           1.125   
3                      0.000000e+00       0.000000           0.000   
4                      3.050000e+00       7.700000          14.850   
5                      1.120000e+00       0.760000           3.060   
6                      1.200000e+00       0.000000           4.500   
7                      0.000000e+00       0.000000           1.700   

   alpha-Bisabolol  2-Hexyl-1-decanol  cluster  
0         0.000000                0.0        0  
1         2.333333                0.0        1  
2         0.000000                0.0        2  
3         0.000000                1.3        3  
4         0.000000                0.0        4  
5         0.540000                0.0        5  
6         0.000000                0.0        6  
7         0.000000                0.0        7  

[8 rows x 68 columns]
Please notice that the cluster indices (i.e. names) might be different than in previous cell!
Cluster 0 is mainly influenced by compounds: ['alpha-Pinene', 'Myrcene', '(Z)-beta-Ocimene', 'delta-cadinene', 'ar-Curcumene']
Cluster 1 is mainly influenced by compounds: ['gamma-Muurolene', 'alpha-Zingiberene', 'alpha-Eudesmol', 'delta-cadinene', 'gamma-Cadinene']
Cluster 2 is mainly influenced by compounds: ['delta-cadinene', 'gamma-Muurolene', '(E)-Caryophyllene', 'gamma-Cadinene', 'alpha-Copaene']
Cluster 3 is mainly influenced by compounds: ['gamma-Eudesmol', 'gamma-Cadinene', 'gamma-Muurolene', 'Isolongifolol', 'Isolongifolol methyl ether']
Cluster 4 is mainly influenced by compounds: ['alpha-Eudesmol', 'beta-Eudesmol', 'alpha-Pinene', '(E)-Nerolidol', '(E)-beta-Ocimene']
Cluster 5 is mainly influenced by compounds: ['gamma-Muurolene', 'delta-cadinene', 'gamma-Cadinene', 'Germacrene D', '(E)-Caryophyllene']
Cluster 6 is mainly influenced by compounds: ['Germacrene D', 'alpha-Zingiberene', 'delta-Amorphene', 'gamma-Muurolene', '(E)-Caryophyllene']
Cluster 7 is mainly influenced by compounds: ['(E)-Caryophyllene', 'alpha-Guaiene', 'delta-cadinene', 'gamma-Cadinene', 'gamma-Muurolene']

Samples in each cluster:
Cluster 0 contains samples: ['G', 'H', 'I']
Cluster 1 contains samples: ['B', 'C', 'D']
Cluster 2 contains samples: ['O', 'Q', 'R', 'S']
Cluster 3 contains samples: ['P']
Cluster 4 contains samples: ['E', 'F']
Cluster 5 contains samples: ['J', 'K', 'L', 'M', 'N']
Cluster 6 contains samples: ['A']
Cluster 7 contains samples: ['T']
```

In [12]:

```
transposed_centroids_df = centroids_df.transpose()

# Save the transposed DataFrame to a CSV file
transposed_centroids_df.to_csv('transposed_centroids.csv')
```
